# Potential Use of Marine Plants as a Source of Bioactive Compounds

**DOI:** 10.3390/molecules30030485

**Published:** 2025-01-22

**Authors:** Maria del Mar Ribas-Taberner, Pere Miquel Mir-Rossello, Lorenzo Gil, Antoni Sureda, Xavier Capó

**Affiliations:** 1 Research Group on Community Nutrition & Oxidative Stress, University of the Balearic Islands-IUNICS, E-07122 Palma de Mallorca, Mallorca, Spain; m.ribas@uib.cat (M.d.M.R.-T.); antoni.sureda@uib.es (A.S.); 2 Interdisciplinary Ecology Group, Department of Biology, University of the Balearic Islands, E-07122 Palma, Balearic Islands, Spain; pere-miquel.mir@uib.cat; 3 Research Group on Plant Biology Under Mediterranean Conditions, Departament de Biologia, Universitat de les Illes Balears (UIB)-Agro-Environmental and Water Economics Institute (INAGEA), E-07122 Palma, Balearic Islands, Spain; lorenzo.gil@uib.es; 4 CIBEROBN (Physiopathology of Obesity and Nutrition), Instituto de Salud Carlos III, E-28029 Madrid, Spain; 5 Community Nutrition & Oxidative Stress, Research Group, Health Research Institute of Balearic Islands (IdISBa), E-07120 Palma de Mallorca, Mallorca, Spain; 6 Translational Research in Aging and Longevity (TRIAL) Group, Health Research Institute of the Balearic Islands (IdISBa), E-07120 Palma de Mallorca, Mallorca, Spain

**Keywords:** marine plants, antioxidant, polyphenols, bioactive compounds, plant extracts

## Abstract

The search for bioactive natural compounds, traditionally focused on terrestrial environments, has increasingly expanded to the seas and oceans, opening new frontiers for exploration. Among the diverse organisms inhabiting these ecosystems, marine phanerogams have emerged as a promising source of health-promoting bioactive compounds. This review highlights the distinctive chemical diversity of seagrasses including species such as *Posidonia oceanica*, *Zostera marina*, and *Cymodocea nodosa,* among others, and focusses on the growing interest in natural therapies as alternatives to conventional pharmaceuticals. Compounds such as polysaccharides or secondary metabolites such as polyphenol and flavonoids produced by marine plants exhibit a broad range of beneficial properties, including anti-inflammatory, antibacterial, antioxidant, and antidiabetic qualities. This review describes how these compounds can mitigate inflammation, promote skin health, and combat oxidative stress. Moreover, certain marine extracts have demonstrated potential to inhibit cancer cell growth and improve metabolic disorders like obesity and diabetes. The manuscript also discusses the potential of marine plant extracts in the development of novel therapeutic agents to address various illnesses, including infections, chronic diseases, and metabolic disorders. It emphasizes the need for further research to fully elucidate the mechanisms underlying the activity of these bioactive compounds and their potential therapeutic applications. In summary, this study highlights marine plants as a valuable reservoir for identifying organic molecules, paving the way for innovative advancements in medical and healthcare interventions.

## 1. Introduction

The growing interest in ethnomedicine has significantly driven the search for new drugs and biologically active compounds derived from plant organisms over recent decades [1]. This interest stems largely from the undesirable side effects associated with many conventional drugs, prompting the exploration of alternative therapeutic sources. Among plant organisms, marine plants, particularly seagrasses, represent a valuable reservoir of natural compounds with highly diverse chemical and structural characteristics [2,3,4]. Although it is well established that certain marine plants have been traditionally employed in natural remedies to treat muscle pain, wounds, gastrointestinal issues, and skin diseases, research into their specific active compounds remains limited [5]. However, recent years have witnessed a notable acceleration in the study of bioactive compounds derived from marine organisms, with the aim of developing novel dietary supplements and pharmaceutical agents.

Marine phanerogams, flowering plants fully adapted to life in marine environments, are distributed primarily along coastal zones of the world’s seas and oceans [6]. These plants belong to four main botanical families: Hydrocharitaceae, Zosteraceae, Cymodoceaceae, and Posidoniaceae, comprising approximately six principal genera. The most notable genera include *Thalassia*, *Halophila*, and *Enhalus* (Hydrocharitaceae); *Zostera* and Phyllospadix (Zosteraceae); *Cymodocea*, *Halodule*, and *Syringodium* (Cymodoceaceae); and *Posidonia* (Posidoniaceae). Collectively, these families encompass 72 recognized species of marine phanerogams [7].

Marine phanerogams play an essential role in the food web of their habitats, providing numerous ecosystem services that are important to humans, especially for populations that occupy coastal areas. These include oxygenation of marine waters, carbon sinks, nutrient balance, coastal protection and nutrient supply to coastal dunes [8,9]. Moreover, marine plants produce a wide array of secondary metabolites that allow them to withstand various ecological stresses [10]. These include primary metabolites such as polysaccharides and secondary metabolites such as polyphenolic substances, peptides and others. Polyphenolic compounds, characterized by their hydroxylated aromatic rings, encompass a diverse range of molecules, such as flavonoids, phenolic acids and phenolic terpenoids [11,12]. These metabolites exhibit distinctive chemical structures and a broad spectrum of biological activities, including antioxidant, anti-inflammatory, antimicrobial, antidiabetic, and anticancer effects, underscoring their potential as therapeutic agents [13]. Of particular interest are natural compounds with antioxidant properties, which have garnered attention for their free radical scavenging capabilities [14]. Similarly, plant-derived antimicrobial agents have gained prominence due to growing concerns over the limitations of conventional antibiotics and synthetic food preservatives [15,16].

**Figure 1:**
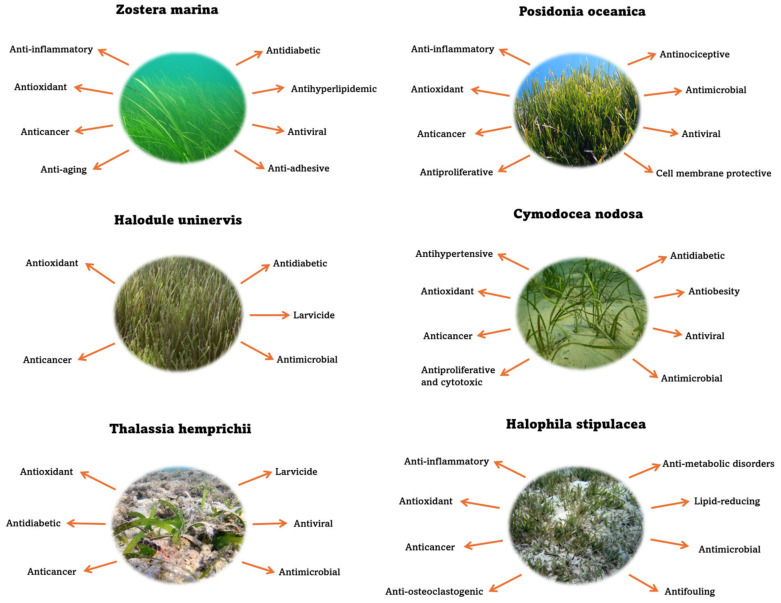
Potential bioactive properties of marine plants.

In this review, we examine the bioactive compounds isolated and identified from six marine plant species: *Cymodocea nodosa*, *Halodule uninervis*, *Halophila stipulacea*, *Posidonia oceanica*, *Thalassia hemprichii*, and *Zostera marina* selected within each family for having greater availability of data in the bibliography. Additionally, we discuss their biological properties, mechanisms of action, and potential applications in therapeutic development (see Figure 1).

## 2. Marine Phanerogams and Bioactive Compounds 

### 2.1. Posidonia oceanica (Delile) (Posidoniaceae)

*Posidonia oceanica*, a marine phanerogam unique to the Mediterranean Sea, forms extensive meadows spanning over 50,000 km^2^. It thrives at depths of 0 to 40 m along the coastal regions. These meadows are essential to marine ecosystems, acting as a key oxygen source for the Mediterranean coast. Furthermore, *P. oceanica* serves as a crucial breeding and nesting site for various economically valuable species, provides habitat and nourishment for marine life at different stages of development, and plays a significant role in safeguarding the coastline from erosion [17,18,19]. This marine phanerogam is characterized by long, strap-like leaves, which can grow up to 1 m in length and are arranged in dense clusters. *P. oceanica* presents rhizomes that spread horizontally beneath the sediment. This rhizomatic growth allows the plant to reproduce vegetatively, forming large meadows that can cover significant areas of the seabed [20,21].

Several studies have identified numerous bioactive compounds in *P. oceanica*. In this regard, polyphenols (such as flavonoids and phenolic acids), tannins, alkaloids, sesquiter-penes, organic acids, and fatty acids, all with bioactive properties, have been found in *P. oceanica* extracts [22,23,24]. These compounds exhibit a wide range of biological properties, including antioxidant, anti-inflammatory, antimicrobial, antiproliferative, and anticancer activities [22,25,26]. Extracts of *P. oceanica* can be prepared using various solvents, such as water, methanol, ethanol, acetone, and non-polar organic solvents like hexane and chloroform. Methanol is commonly used to extract phenolic compounds, particularly in antioxidant research. Due to its lower toxicity and its ability to extract phenolics, 70% ethanol is also used for phenolic compound extraction [27]. On the other hand, tannin extraction is enhanced with a mixture of acetone and water [27,28]. For water-soluble compounds, water or mixtures of water with ethanol and methanol are commonly used [28]. Lipophilic compounds, such as terpenes, are extracted using non-polar solvents like hexane or chloroform [29,30].

Among the bioactive compounds, phenolic compounds are one of the most important substances targeted in plant extracts due to their strong antioxidant capacity, which is often linked to their abundance in plants [31]. The majority of the previously published investigations on the phenolic content of *P. oceanica* leaves reveal notable differences in the quantity, composition, and concentration of the various constituents, probably derived from different methodologies applied and forms/periods of sample collection [32]. In general, in all studies, chicoric acid is the most abundant polyphenol isolated from *P. oceanica*. Additionally, significant amounts of caftaric acid, gentisic acid and ferulic acid have also been identified in *P. oceanica* extracts [32]. Leri and collaborators reported that the most abundant polyphenolic compound in a hydrophilic extract of *P. oceanica* was catechin, followed by epicatechin, ferulic acid, and chlorogenic acid, although in lower proportions [22,33]. Another study has analysed differences in the content of phenolic compounds in fresh and dried samples of *P. oceanica*, and their corresponding antioxidant capacities [27]. In this study, researchers reported the presence of nine different phenolic compounds (phloroglucinol, gallic acid, hydroxybenzoic acid, vanillic acid, caffeic acid, coumaric acid, ferulic acid, chicoric acid and quercetin) and their concentration varied between fresh and dry *P. oceanica* leaves, with dried leaves showing higher concentrations of phenolic compounds [27]. Additionally, the antioxidant properties of both fresh and dried *P. oceanica* leaves were evaluated. While both types of leaves exhibited high antioxidant potential, the dried leaves demonstrated significantly greater antioxidant activity [27].

The antioxidant capacity of *P. oceanica* extracts rich in polyphenols has been demonstrated in cell cultures against UV radiation exposure. A study performed using a hydroalcoholic extract from *P. oceanica*, composed mainly of chicoric acid, evidenced high radical scavenging activity, lipolytic activity and relevant capability to increase fibroblast growth and collagen synthesis, demonstrating the skin-protective properties of this hydroalcoholic extract [34]. Regarding skin protection, it was evidenced that a hydroalcoholic extract of *P. oceanica* leaves rich in polyphenols inhibited psoriatic dermatitis induced by topical administration of imiquimod in mice [25]. These effects were probably associated with a reduction in the plasma levels of proinflammatory cytokines as tumour necrosis factor alpha (TNFα), interleukin (IL)-17 and interferon gamma (IFNγ). The photo protective effect of *P. oceanica* extracts was also evidenced in a study pre-treating fibroblasts with *P. oceanica* extracts resulting in increased cell viability after ultraviolet (UV) light exposition [27].

Researchers from the University of Thessaloniki studied the anticancer effects of hydroalcoholic extracts of *P. oceanica* leaves containing thirty-seven phenolics. The major phenolic compound found in this extract was chicoric acid and at lower levels, epigallocatechin gallate, p-coumaric acid, rutin hydrate, sinapinic, caftaric, ferulic and trans-cinnamic acids were also detected in the extracts [26]. The extracts evidenced significant antiproliferative effects against LS174 colon cancer cells [26]. Similarly, it was evidenced that the hydrophilic extract from *P. oceanica* was able to reduce the motility of the highly invasive HT1080 fibrosarcoma cell line, probably through a reduction in the expression of gelatinases and inhibiting gelatinolytic activity [33]. Another study using a hydroalcoholic extract of *P. oceanica* leaves, composed mainly of catechins, gallic acid, ferulic acid, epicatechin, and chlorogenic acid avoided intracellular lipid accumulation and blocked the mitogen-activated protein kinase (MAPK)/nuclear factor kappa B (NF-κB) axis, reducing metalloproteinase 2 and 9 in an in vitro model of hepatocarcinoma HepG2 cells [35].

The antidiabetic effects have been investigated after an extraction of secondary metabolites from the rhizome of *P. oceanica* conducted with butanol. The extraction allowed the isolation of 32 phenolic acids, 17 cinnamic acids and 29 flavonoids, with p-hydroxybenzoic acid, protocatechuic acid, epi-catechin, syringic acid and catechin being the most relevant compounds [30]. This extract evidenced potent antidiabetic activity in vitro with α-glucosidase inhibitory activity but also in a rat model of diabetes induced by streptozotocin [30]. The extract improved the pathological metabolic state and biochemical parameters of the diabetic rats evidenced by improved fasting blood glucose, insulin levels, homeostasis model assessment of insulin resistance (HOMA-IR) and glucose transporter 4 (Glut 4) [30].

Research has demonstrated that *P. oceanica* extracts possess antibacterial properties effective against both Gram-positive and Gram-negative bacteria, with notable activity against *P. aeruginosa* and *S. aureus* [29]. Additionally, *P. oceanica* has exhibited antiviral potential; notably, in 2018, Farid et al. reported that extracts from *P. oceanica* balls reduced H5N1 virus infection by 45% [36]. Furthermore, polypeptide-enriched fractions derived from the rhizomes and leaves of the seagrass have been reported to exhibit antimicrobial activity [37]. These effects were observed against Gram-positive bacteria (*Staphylococcus aureus*, *Enterococcus faecalis*), Gram-negative bacteria (*Pseudomonas aeruginosa*, *Escherichia coli*), and the yeast *Candida albicans*.

In addition to phenolic chemicals, polysaccharides have also been linked to the bioactive qualities of extracts from marine plants including *P. oceanica* [23,38]. The extraction of polysaccharides present in *P. oceanica* is a lengthy process that often requires high temperatures, the use of specific enzymes, ultrasound, and microwaves [39,40]. Compounds such as sulfated polysaccharides (including galactans, fucans and xyloglucans), pectin, alginates and lignans have been extracted from *P. oceanica* extracts [39,41]. The main polysaccharide isolated from *P. oceanica* is cellulose; however, it is a polysaccharide without interesting biological activity properties [41]. It have evidenced that polysaccharide or carbohydrates from *P. oceanica* rich in galactose, glucose, arabinose, rhamnose and xylose presented both anti-inflammatory and antinociceptive effects in vivo [42]. An interesting study reported that the lignin water-soluble fraction extracted from *P. oceania* egagropili (sea balls) has notable antioxidant activity [40]. This activity demonstrated remarkable stability over a period of six months and could be seamlessly integrated into a protein-based film, allowing for its gradual release over time (see Table 1).

### 2.2. Zostera marina *L.* (Zosteraceae)

*Z. marina* is a rhizomatous, monoecious species with nodes that bear 1–2 root fascicles and a brachyblast producing 3–8 leaves. Leaves are linear, reaching up to 75 cm in length and occasionally extending to 1 m, with rounded apices and a fused sheath at the base. Reproductive shoots feature numerous leaves and inflorescences. The inflorescences are spadices with 9–20 hermaphroditic, aperianthic flowers, each containing a single sessile stamen and a sessile, monocarpellary, conical ovary. The fruits are ellipsoidal to ovoid achenes, measuring 2.5–4 × 2–3 mm [43,44]. *Z. marina* forms seagrass meadows in estuaries, marshes, and seabeds, generally down to depths of 20 m, and is found across most coastal areas of the Northern Hemisphere [44].

Studies on the components of *Z. marina* extracts have led to the isolation and identification of various metabolites with significant bioactive properties, highlighting unsaturated fatty, phenolic compounds such as rosmarinic acid, luteolin, sagerinic acid, umbelliferone and pectins, particularly zosterin (4,2′-dihydroxy-4′,6′-diacetoxydihydrochalcone 2′-O-glucoside) [45,46]. An interesting study that involved supercritical CO_2_ extraction followed by mass spectrometric characterization showed the presence of 77 different biologically active components in *Z. marina*, 53 of which were polyphenols [47]. Furthermore, similarly to what happened with P. oceanica, its composition varies significantly depending on the country and among different sites within countries [48]. These compounds exhibit activities of interest for human health, including antioxidant, anti-inflammatory, antiviral, antidiabetic, antihyperlipidemic, anti-aging, and anticancer effects.

In this sense, both water and methanol extracts of *Z. marina*, rich in polyphenols, have repeatedly demonstrated strong antioxidant activity [49,50,51]. Metabolites such as zosterin and pectin have shown superior antioxidant activity compared to some commercially available antioxidant compounds [49,50]. Notably, molecules like rosmarinic acid and luteolin, which are also present in terrestrial plants such as artichoke (*Cynara scolymus* L.) and rosemary (*Salvia rosmarinus* Spenn), exhibit potent antioxidant properties [45,50]. In particular, luteolin holds promise for the development of anti-aging products. Specifically, luteolin, isolated from the ethyl acetate extract of *Z. marina*, demonstrated higher matrix metalloproteinase-1 (MMP-1) inhibitory activity than retinol in human skin fibroblasts [50]. Additionally, luteolin suppressed the expression of IL-1α and IL-6, highlighting its anti-inflammatory potential. Another study also reported significant anti-phototoxicity and anti-melanogenesis activities of the methanolic extract of *Z. marina* increasing the resistance of HaCaT cells to UVB radiation and reducing melanin synthesis in B16 melanoma cells [52].

*Z. marina* also produces an anti-adhesive compound known as p-sulfoxy-cinnamic acid, or zosteric acid [53]. This compound has been reported to inhibit the colonization of its leaf surfaces by various organisms, making its mechanism of action particularly interesting for the development of antiviral drugs [53]. Both zosteric acid and its derivatives have demonstrated antiviral activity against the Dengue virus [53]. Interestingly, this mechanism does not prevent virus–cell binding; rather, it strengthens this binding. This observation has led to the hypothesis that zosteric acid’s antiviral effect may arise from binding or trapping the virus on the target cell, thereby preventing its spread to other cells. In this sense, a computational docking analysis reported that zosteric acid is capable of binding to non-structural protein 5 (NS5), which plays a crucial role in the replication of Dengue virus [54].

In addition, the polyphenol components in *Z. marina* extracts have shown antidiabetic and antihyperlipidemic effects, primarily attributed to rosmarinic acid, luteolin, and 7,3′-disulfate luteolin (DSL) [55]. *Z. marina* extracts, either extracted in polar or non-polar solvent, exerted lipase inhibitory activity in in vitro assays [56]. Pharmacological studies in animal models of type 2 diabetes and hyperlipidaemia have revealed that DSL exhibits greater antidiabetic and antilipidemic activity than luteolin, highlighting its potential as a therapeutic agent [57]. The use of these flavonoid polyphenols could facilitate the development of novel therapies for preventing and treating metabolic disorders. However, further research is required to fully elucidate their mechanisms of action. Styshova and collaborators proposed potential mechanisms for these polyphenol compounds, suggesting that rosmarinic acid, luteolin and their analogues may interfere with PPAR activity, thereby enhancing insulin sensitivity and exerting anti-inflammatory effects. Moreover, DSL is hypothesized to possess superior bioavailability and the ability to modulate lipoprotein metabolism, potentially conferring an anti-atherosclerotic effect [55].

Regarding anticancer activity, diverse phenolic compounds such as chicoric, p-coumaric, rosmarinic, benzoic, ferulic, and caffeic acids, found in the ethanolic extract of *Z. marina*, have demonstrated cytotoxic activity against various cancer cell lines, including human breast adenocarcinoma, colon, cervix, prostate adenocarcinoma and neuroblastoma [58]. In this study, supercritical CO_2_ extracts were more active than Soxhlet extracts with IC50 values of 25, 20 and 8 μg/mL in neuroblastoma, colon and cervix cancer cell lines. Among the isolated compounds, p-coumaric acid exhibited selective cytotoxic activity in colon and cervical cancer cells. Additionally, the diarylheptanoids identified in *Z. marina* showed selective cytotoxic effects on HCT116 tumour cells with IC50 3.6 μM at 48 h [59]. In this sense, zosterabisphenone B induced apoptosis in HCT116 colon cancer cells increasing the levels of cleaved caspases, Poly (ADP-ribose) polymerase (PARP) and BH3 Interacting Domain Death Agonist (BID) proteins and a decrease in Bcl-2 and c-Myc proteins [60]. In a xenograft model, the compound was also capable of reducing the tumour growth. These findings highlight the potential of *Z. marina* bioactive compounds as promising candidates for the development of novel anticancer drugs, particularly for colorectal cancer.

In conclusion, the bioactive compounds identified in *Zostera* species, particularly in *Z. marina*, demonstrate a wide range of health-promoting activities, making them valuable resources for the development of new therapeutic drugs. These compounds show promise for treating metabolic disorders and cancers. However, further research is required to fully elucidate their mechanisms of action and evaluate their therapeutic potential in humans (see Table 2).

### 2.3. Cymodocea nodosa (Ucria) Asch. (Cymodoceaceae)

*C. nodosa* is a rhizomatous, dioecious species characterized by rhizomes with internodes ranging from 1.2 to 16 cm in length. Each node produces 3–4 (occasionally up to 7) erect, serrulate leaves, 16–60 cm in length, with 2–12 cm long sheaths and numerous foliar scars at the base. Male flowers are borne on pedicels of 4–7 (up to 10) cm, each containing two anthers marked by reddish spots [61]. Female flowers are aperianthic, either sessile or sub-sessile, with a bicarpellary ovary and two filiform stigmas. The fruit is an ovoid, crested drupe, measuring 12–14 × 8–10 mm. This species typically inhabits sandy and rocky substrates at depths of up to 30 m, occasionally reaching 70 m. Predominantly found in the Mediterranean, it also occurs along parts of the Atlantic coast, including the southern Iberian Peninsula, Macaronesia, and the western coast of tropical Africa [62].

**Figure 2:**
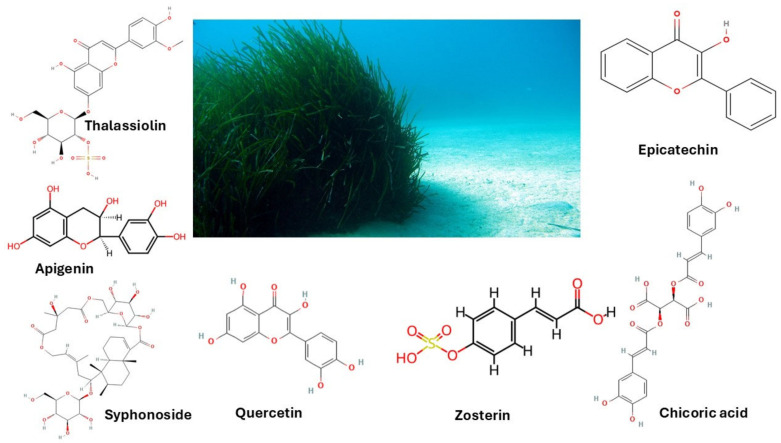
Most representative compounds from marine plants. Thalassiolin from *T. hemprichii;* Apigenin from *H. uninervis*; Syphonoside from *H. stipulacea;* Quercetin from *C. nodosa*; Zosterin from *Z. marina*; Chicoric acid from *P. oceanica*.

Available studies on the potential therapeutic uses of *C. nodosa* focus on the presence of sulphated polysaccharides, although it also contains other compounds with therapeutic interest such as phenolics and alkaloids [63]. The first characterization of the sulphated polysaccharide from *C. nodosa* (CNSP) was conducted by Ben Abdallah Kolsi et al. in 2015 [64]. They isolated CNSP via hot water extraction and analysed its chemical composition. The obtained sulphated polysaccharide-enriched fraction was found to have a high content of sulphate and carbohydrates, with proteins present in moderate amounts and lipids in lower concentrations. Further analyses indicated that CNSP also contains 11.03% uronic acid. Preliminary structural studies suggest that the backbone of CNSP consists of branched 6-O-sulfated (1→4)-galactosidic linkages. In general, bioactive polysaccharides function through non-covalent interactions with target proteins, inducing conformational changes that make them valuable for inhibiting or modulating the activity of enzymes involved in specific pathologies. In the case of CNSP, studies have demonstrated its antioxidant capacity and reducing power, both of which are concentration-dependent [65]. These antioxidant properties are attributed to CNSP’s redox capabilities, which are essential for neutralizing and absorbing free radicals, decomposing peroxides, and quenching singlet and triplet oxygen species. Additionally, the presence of sulphate groups and uronic acid in its structure confers CNSP a notable chelating capacity [66] (see Figure 2).

Among the most interesting bioactive properties of CNSP are its anti-obesity and anti-diabetic effects. In 2015, Abdallah et al. demonstrated that CNSP can inhibit pancreatic, intestinal, and serum lipases, as well as intestinal and plasma α-amylases, in high-fat diet (HFD) rats [64]. This inhibition led to reductions in total cholesterol (TC), triglycerides (TGs), and LDL-C levels, while increasing HDL-C levels and decreasing blood glucose levels. Furthermore, the reduction in blood glucose was enhanced by the stimulation of pancreatic β-cells, resulting in increased insulin production. This antilipidemic, anti-cholesterolemic, and antihyperglycemic activity also contributed to improvements in pancreatic, hepatic, and renal functions, as well as a reduction in body weight in rats fed a high-cholesterol diet. In addition, the antihypertensive potential of CNSP has been investigated, revealing that this polysaccharide can inhibit the angiotensin-converting enzyme (ACE) [65]. This property makes CNSP a promising candidate for the development of medications aimed at treating hypertension.

Due to growing concerns over the issues associated with conventional antibiotics and food preservatives, the antibacterial and antifungal properties of CNSP have also been investigated. It has been observed that CNSP binds to bacterial ribosomes, inducing codon decoding errors during mRNA translation, which leads to the synthesis of abnormal proteins and ultimately cell death [66]. Additionally, in Gram-positive bacteria, components of CNSP come into direct contact with the phospholipids in the cell membrane bilayer, increasing ionic permeability (and facilitating CNSP passage) and disrupting intracellular enzymatic systems. In contrast, the lower susceptibility of Gram-negative bacteria to CNSP can be attributed to the reduced diffusion capacity of CNSP through the outer membranes surrounding their cell walls. Further investigation into the antimicrobial activity of purified CNSP compounds is warranted, as they may prove more potent than the complex mixture of CNSP typically used in pure antibiotics and antifungals. Similarly, the antiproliferative and cytotoxic effects of CNSP have been explored. Studies on tumor cell lines have shown that the highest cytotoxicity occurs at a concentration of 0.5 mg/mL.

The bioactive properties of CNSP have also garnered interest in fertility research. In this sense, it has been demonstrated that CNSP has a protective effect against testicular toxicity induced by lambda-cyhalothrin (LTC) [67]. The antioxidant capacity of this sulphated polysaccharide helps mitigate oxidative stress in the testes, resulting in improved semen quality, including enhanced sperm count, motility, and viability, in male rats.

In addition to CNSP, the hydroalcoholic extract of *C. nodosa* (CNE) also exhibits notable bioactive properties. CNE has significant antioxidant activity, which is primarily attributed to its high polyphenol content, especially flavonoids [68]. In fact, an ultrasound-assisted extraction evidenced a high phenolic content of 113 mg gallic acid equivalents/g dry weight, with significant levels of sinapic acid, myricetin, and quercetin-3-O-rutinoside [69]. CNE’s antioxidant potential has attracted interest as a promising component in developing new therapies for metabolic and inflammatory diseases such as diabetes mellitus and obesity. Its antioxidant activity has shown protective effects on pancreatic β-cells, preventing damage and cell death in alloxan-diabetic rats [70]. This protection leads to reduced blood glucose levels, along with decreases in AST, ALT, LDH, LDL-C, TC, and TG, while increasing HDL-C levels. CNE’s partial inhibitory effect on plasma and intestinal α-amylase further supports metabolic regulation by reducing glucose release during starch digestion. Additionally, CNE has demonstrated potential in reversing tissue damage and other oxidative stress complications in the liver, pancreas, and kidneys. Furthermore, studies have revealed that CNE has an inhibitory effect on ACE, making it a promising candidate for the development of antihypertensive medications [68]. Regarding the antibacterial activity of the organic phase, it has been reported that two diarylheptanoids, a meroterpenoid, and a new briarane diterpene exerted notable effects against multidrug-resistant pathogens including *Staphylococcus aureus*, *Mycobacterium phlei*, *Mycobacterium smegmatis*, and *Mycobacterium fortuitum* [71].

Overall, the bioactive properties of both CNSP and CNE hold promising potential for the treatment of metabolic and inflammatory diseases, such as obesity and diabetes mellitus. Their antibacterial and antifungal properties suggest they may be valuable in developing alternatives to conventional antibiotics. Additionally, we emphasize the antiproliferative and cytotoxic effects of CNSP, which could support the advancement of new oncological therapies. Further research into CNSP’s potential to enhance fertility is also warranted, as it may contribute to the future development of treatments for infertility (see Table 3).

### 2.4. Halodule uninervis (Forssk.) Boiss. (Cymodoceaceae)

This rhizomatous, dioecious species has rhizomes with internodes up to 5 cm in length. From each node, 2–4 linear leaves with a tridentate apex emerge, reaching sizes of up to 150 mm in length and 0.25–5 mm in width. A persistent leaf sheath up to 3.5 cm in length surrounds the base, from which inflorescences arise. Each male flower has two stamens on peduncles 1–2 cm in length, with red-coloured anthers approximately 0.5 mm long. Female flowers have an apical style up to 4 mm in length and a bicarpellary gynoecium. The fruit is a drupe measuring up to 2.5 mm [72]. This species forms seagrass meadows in coastal lagoons and reef habitats at depths from 0 to 20 m, distributed along the coasts of the Pacific and Indian Oceans, from the Red Sea to French Polynesia [72].

The preliminary phytochemical screening of *H. uninervis* revealed that this species is rich in secondary metabolites, including phenolic acids, flavonoids, quinones, tannins, terpenoids, and steroids [73]. It is notable that *H. uninervis* is rich in phenolic compounds that confer the plant with self-defense mechanisms. The main phenolic compounds identified in methanolic and ethanolic extracts of *H. uninervis* were caffeic acid, chlorogenic acid, gallic acid, and p-hydroxybenzoic acid, varying their composition depending on the type of extract [74]. In addition, the extracts also contained three flavonoids, including apigenin, apigenin-7-O-glucoside, quercetin, kaempferol, and naringenin. However, in most works investigating the potential biological effects, no in-depth investigation has been made into determining the compounds present in the extracts.

The methanolic and ethanolic extracts of *H. uninervis* have been reported to exhibit strong antioxidant activity by scavenging both the DPPH and the ABTS radicals [75]. The antioxidant effects were mainly attributed to the presence of polyphenols such as apigenin and kaempferol. In further testing with the DPPH assay, comparing different fractions, the fraction of unsaponifiable matter exerted the highest activity respect to the methanol/chloroform extract or phenolic fractions. This was attributed to the presence of a significant amount of the known antioxidant dibutylhydroxytoluene [74].

*H. uninervis* extracts have demonstrated notable antimicrobial activity, particularly against bacteria. These studies have determined the general polyphenol content, but have not delved into their composition, making it difficult to determine the main bioactive compounds. Specifically, methanolic extracts have proven more effective against Gram-positive bacteria, such as *Bacillus subtilis* and *Listeria monocytogenes*, compared to their activity against Gram-negative bacteria like *Aeromonas hydrophila* and *Vibrio harveyi* [76]. Another study showed that ethanolic extracts were effective against *Bacillus cereus*, a Gram-positive bacterium, as well as against *Proteus vulgaris*, a Gram-negative bacterium [77]. This same study demonstrated the extract’s ability to inhibit the growth of the fungus *Cryptococcus neoformans*. This study reported after gas chromatography mass analysis the presence of the extract of neophytadiene, a terpenoid with antimicrobial properties. Various organic extracts of the plant have also shown efficacy against other Gram-positive bacteria, including *Staphylococcus aureus* and *Micrococcus luteus*, as well as against Gram-negative bacteria such as *Escherichia coli*, *Klebsiella pneumoniae*, *Pseudomonas aeruginosa*, and *Salmonella typhi* [78].

Various studies have analysed the antilarvicidal effects of different extracts from *H. uninervis*. The effects of ethyl acetate extract on Culex pipiens and methanolic extract on Aedes aegypti have been observed [79,80]. In the latter study, a green synthesis of silver nanoparticles containing *H. uninervis* aqueous leaf extract was also developed, which increased toxicity, reducing the LC50 value from 295.6 ppm for the methanolic extract to 12.5 ppm for the nanoparticles [79].

Another interesting function of *H. uninervis* extracts is their potential antidiabetic activity. Ethanolic and ethyl acetate extracts have shown in vitro inhibitory activity against the enzyme α-glucosidase [81]. The results showed a greater inhibitory capacity for the ethanolic extract with an IC_50_ of 74.9 ppm. In an intriguing study using a Streptozotocin-induced diabetic mouse model, a metabolic extract of the plant (administered at 150 and 250 mg/kg/day for 18 days) was able to reduce blood glucose levels and animal weight, as well as restore reduced white blood cell counts [82]. The study also reported improvements in liver and kidney function following treatment with *H. uninervis* extracts, demonstrated by significant decreases in alkaline phosphatase (ALP), glutamate pyruvate transaminase (GPT), blood urea nitrogen (BUN), and creatinine levels compared to the diabetic group, along with a reduction in hepatic markers of oxidative stress.

Finally, for this plant species, the anticancer activity of some extracts on different cell lines is noteworthy. However, there are still no studies on animal models to confirm the results obtained in vitro. The crude extract, after 72 h exposure, showed a significant cytotoxic effect against human SKOV-3 ovarian and MCF-7 breast carcinoma cells, with IC50 values of 0.52 and 2.3 μg/mL, respectively [74]. Among other fractions tested, the chloroform fraction exhibited notable activity against DU-145 prostate cancer cells (IC50 10.4 μg/mL) and PANC-1 pancreatic cancer cells (IC50 8.6 μg/mL). Additionally, the non-saponifiable fraction demonstrated cytotoxicity against MCF-7 cells (IC50 3.7 μg/mL), while the saponifiable fraction (free fatty acids) was active against DU-145 prostate cells (IC50 20.2 μg/mL) and HeLa cervical cancer cells (IC50 4.6 μg/mL). In another study, the ethyl acetate fraction of *H. uninervis* was investigated against A549 (lung carcinoma), HeLa, HT29 (colorectal adenocarcinoma) and A375 (malignant melanoma) cells [83]. The results evidenced cytotoxic effects of the extract after 24 h with an IC50 of 91.6 ± 1.6 μg, 269 ± 5 μg, 845 ± 36. μg and >1000 µg dry extract, respectively (16). A more detailed study on the most sensitive cells, A549, showed that the extract induced apoptosis with notable morphological changes and the appearance of ladder-like DNA. The anticancer activity of *H. uninervis* ethanolic extract (0–400 μg/mL for 72 h) has also been investigated against MDA-MB-231 triple-negative breast cancer cells [75]. The results reported significant anti-proliferative and anti-metastatic effects, which were associated with the downregulation of the STAT3 (signal transducer and activator of transcription 3) signalling pathway (see Table 4).

### 2.5. Thalassia hemprichii (Ehrenb. ex Solms) Asch. (Hydrocharitaceae)

This rhizomatous, dioecious species has rhizomes that typically contain air channels and exhibit triangular scars at the nodes. Each node produces 2–6 leaves, surrounded at the base by persistent sheaths that are 3–8 cm in length. The falcate leaves measure 7–25 (up to 40) cm in length and 0.5–1 cm in width, with red-violet spots or streaks, rounded apices, and margins that are entirely or slightly serrated [84]. Male flowers are grouped, and each is accompanied by a single bract, containing an androecium with 3–12 stamens. Female flowers are solitary and subsessile, featuring a hexacarpellary ovary with six free styles up to 7 mm long. The fruit is a globular, spiny berry, measuring 2–2.5 × 1.5–3.2 mm [84]. This species forms seagrass meadows in shallow subtidal zones, typically up to 10 m in depth, and is distributed along the coasts of the Pacific and Indian Oceans, from the Red Sea to French Polynesia. Its range also includes the coasts of Madagascar and southwestern Australia [84].

The bioactive components found in species of the genus *Thalassia* are associated with a range of therapeutic activities, including antimicrobial, antiviral, antioxidant, antidiabetic, anticancer, cytotoxic, and larvicidal effects [85,86,87]. Extracts from *T. hemprichii* can be prepared using various solvents (e.g., acetone, diethyl ether, ethyl acetate, methanol, ethanol, and water), which yield extracts with distinct chemical profiles [88]. Compounds identified in *T. hemprichii* extracts include sulphated flavonoids (including thalassiolins), flavonoid glycosides, sterols, sterolic glycosides, phenolic acids, carotenoids, nitrogenous compounds, and benzophenones [89]. Notably, flavonoids, alkaloids, and tannins are of particular interest.

Among the biological activities of the different extracts, the antimicrobial capacity stands out, with ethanol and acetone extracts exhibiting the strongest effect [86,87,88]. Specifically, flavonoids present in *T. hemprichii* exhibit antifouling, antibacterial, and antifungal properties by altering the permeability of bacterial cell walls, microsomes, and lysosomes [90]. Thalassiolins A–C (luteolin 7-β-D-glucopyranosyl-2′-sulfate, luteolin 7-β-D-glucopyranoside, and luteolin 7-sulfate, respectively) were initially identified in *T. testudinum* but are also present in *T. hemprichii* [91]. Thalassiolin D (luteolin 3′-sulfate), however, has been uniquely characterized in *T. hemprichii*. These compounds are sulphated flavone glycosides with notable biological activities: specifically, thalassiolin A shows antifungal properties, thalassiolins A–C demonstrate antiviral effects against HIV, and thalassiolin B exhibits both antioxidant and skin-regenerative effects. Furthermore, thalassiolin D has shown antiviral activity against hepatitis C virus [91,92]. Alkaloids in *T. hemprichii* extracts function as bacteriostatic agents, with structural characteristics suggesting they may act as DNA intercalators or topoisomerase inhibitors [93]. Additionally, they seem to interfere with bacterial cell wall peptidoglycan, ultimately leading to cell death. Tannins, meanwhile, interact with bacterial cell membranes to inactivate enzymes such as reverse transcriptase and DNA topoisomerase, thereby inhibiting bacterial replication [94]. They also inhibit adhesin enzymes, disrupting protein transport across the cell wall. Interestingly, the endophytic fungi (fungi that reside within the healthy tissues of plants) associated with *T. hemprichii* show significant potential for developing antimicrobial agents, particularly antifungal drugs [86]. Specifically, some ethyl acetate extracts from various isolated endophytic fungi showed minimum inhibitory concentrations (MIC) of less than 10 µg/mL for some of the pathogenic fungi.

Extracts from *T. hemprichii* exhibit strong antioxidant properties that are directly linked to their antimicrobial activity. Both the ethanolic extract and the metabolic extract of *T. hemprichii* demonstrate significant antioxidant activity, with the extraction using 50% ethanol and 1N HCl proving to be the most effective option, leading to an IC50 of 83.5 μg/mL, 41.03 mg GAE/g and 22% yield extract [95].

Regarding the antidiabetic potential of *T. hemprichii*, the ethanolic extract displayed inhibitory activity against alpha-amylase, beta-glucosidase, and pancreatic lipase, correlating this activity with the phenolic metabolites present in the extracts [96]. Additionally, the extract improved GLUT2 and insulin levels while decreasing glucose levels. The ability to mitigate oxidative stress generated by free radicals and dyslipidaemia indicates an antioxidant effect, suggesting a hypoglycaemic and hypolipidemic function [87,96]. Thus, the ethanolic extract of *T. hemprichii* demonstrates potential as an antidiabetic agent for managing calorie intake. Notably, thalassiolins A and C, in addition to their antimicrobial effects, exhibit inhibitory activity against nitric oxide synthase, ROS 1 kinase, human pancreatic lipase, and fat mass and obesity-associated (FTO) proteins, making them of interest for the development of new antioxidants and anti-obesity medications [56].

The larvicidal properties of *T. hemprichii* extracts are also significant. Both the ethanolic and methanolic extracts display larvicidal activity, with the methanolic extract from leaves being the most effective [97,98].

Finally, *T. testudinum* from the same genus is of pharmacological interest. This species is primarily recognized for its anti-inflammatory, antioxidant, and anticancer properties [11]. Studies have shown that the polyphenols in the extract of *T. testudinum* exhibit cytotoxic, antiproliferative, and antimigratory effects on cancer cell lines [99]. These effects were also evidenced in an allograft murine colorectal cancer model with a significant reduction in tumor growth in a process mediated by the activation of the Activating Transcription Factor 4 (ATF4)-p53-NFκB pathway and autophagy [99] (see Table 5).

### 2.6. Halophila stipulacea (Forssk.) Asch. (Hydrocharitaceae)

This rhizomatous, dioecious species features leaves with short petioles, each enclosed by a pair of large, transparent, elliptical scales. The leaf blades are oblong with serrated margins and can grow up to 6 cm in length [100]. Male flowers are borne on short pedicels, accompanied by three basal bracts measuring 5–6 mm, and have a perianth composed of three tepals (3–4 mm long) and an androecium with three stamens. Female flowers are sessile or sub-sessile, with a tricarpellary ovary and styles extending up to 4 cm. The fruit is a drupe measuring up to 5 × 3.5 mm [101]. This species forms seagrass meadows at depths ranging from 3 to 10 m, occasionally extending to 30 m. First described in the Red Sea (Forsskål, 1775), its distribution includes the coasts of East Africa, Madagascar, the Arabian Sea, the Persian Gulf, and the western coast of India [102].

*H. stipulacea* is widely recognized for its production of secondary metabolites with biological activity [12,103]. Previous studies have identified polyphenols and terpenoids as the primary bioactive compounds in *H. stipulacea*. Specifically, the flavonoid apigenin and the glycoterpenoid syphonoside have been reported as the main bioactive compounds isolated from *H. stipulacea* extracts [96,104,105]. Interesting research has investigated the extraction of bioactive compounds from leaves and stem from *H. stipulacea* using different solvents including hexane, ethyl acetate, and methanol [106]. A total of 110 bioactive compounds were identified in the extracts, including vitamins (choline), fatty acids (13-Docosenamide, palmitoylcarnitine), sugars, amino acids, polyphenols (luteolin, spiraeoside, or aloenin), chlorophylls (pheophorbide A, pheophytin), terpenoids (ginkgolide B, perillyl alcohol, xanthomonic Acid), and alkaloids (3-ethyl-2,3,6,7,8,8a-hexahydropyrrolo [1,2-a]pyrazine-1,4-dione) [106].

The antioxidant capabilities of mature and young leaves of *H. stipulacea* have been studied in fibroblasts [105].

It was demonstrated that mature leaves exhibited higher ROS-scavenging activity and were more effective in mitigating oxidative stress-related damage in fibroblast cell lines compared to young leaves. This enhanced activity is likely attributed to the elevated carotenoid content in mature leaf extracts and a greater carbohydrate contribution to organic matter [105].

The bioactive compounds of *H. stipulacea* hold potential as a tool in cancer treatment, as antiproliferative effects have been observed in vitro against various types of cancer, including osteosarcoma, human neuroblastoma, and human colorectal carcinoma. These antiproliferative effects were observed in both hexane and ethyl acetate extracts derived from leaves and stems [106,107]. The observed anticancer (cytotoxic) properties were linked to the presence of luteolin, apigenin and matairesinol in the extract [106].

Extracts from *H. stipulacea* have also been reported to exhibit anti-inflammatory and anti-metabolic disorder activities, likely due to their high polyphenol concentrations [96,108]. However, further research is needed to identify the specific metabolites responsible for these effects. Among the polyphenols with lipid-lowering properties, cirsimarin and spiraeoside were highlighted, along with amino acids such as N-acetyl-L-tyrosine [106].

Additionally, *H. stipulacea* extracts have demonstrated antimicrobial activity against fungi, bacteria, and yeast [107]. For instance, aqueous, hydroalcoholic, chloroform, and ethyl acetate extracts of *H. stipulacea* leaves showed potent antimicrobial effects against a wide range of bacteria, including *Bacillus subtilis*, *Staphylococcus aureus* or *Pseudomonas aeruginosa* [109]. Notably, its antimicrobial activity is associated with a strong antifouling capacity, potentially linked to the presence of compounds like 13-decosenamide and cinnamic acids [106].

Finally, it has been demonstrated that diethyl ether and butanol extracts from *H. stipulacea* exhibit significant anti-osteoclastogenic effects, likely due to the presence of phytosterols in the extracts [110] (see Table 6).

## 3. Conclusions

In conclusion, marine plants represent an underexplored yet highly promising source of bioactive compounds with significant potential for medical applications. Their diverse secondary metabolites, including polyphenols and flavonoids, and some primary metabolites, such as polysaccharides, exhibit a wide range of biological activities such as antioxidant, antibacterial, anti-inflammatory, and anticancer properties. These compounds have demonstrated potential in addressing major health challenges, including cancer, infectious diseases, and metabolic disorders, highlighting their therapeutic value. However, to date, there are very few studies focused on specific compounds found in these plants, such as thalassiolins or zosterin, nor are there any clinical trials using any of the extracts. Despite these encouraging findings, further research is essential to fully elucidate their mechanisms of action, optimize extraction and purification methods, and rigorously evaluate their safety and efficacy in preclinical and clinical settings. Additionally, investigations into the sustainable harvesting and cultivation of marine plants will be crucial to ensure a steady supply of these valuable resources.

## Figures and Tables

**Table 1:** Main compounds and bioactive properties in *P. oceanica*.

Extract Type	Main Compounds	Activity	Experimental Model	Reference
Hydrophilic	Epicatechin and phenolic acids	Invasive reducing activity in cancer cells	Cell culture HT1080 cell line	[33]
Methanolic and ethanolic	Phenolic acids (mainly chicoric acid)	Antioxidant Photoprotective	In vitro HS-68 cell line	[27]
Hydroalcoholic	Chicoric acid	Radical scavenging Lipolytic activity Skin protection	In vitro Human dermal fibroblast	[34]
Hydroalcoholic	Not determined	Anti-inflammatory Skin protection	In vivo C57BL/6 mice	[25]
Methanolic	Chicoric acid, epigallocatechin gallate and p-coumaric acid	Antiproliferative Antioxidant	In vitro LS174 cell line	[26]
Buthanolic	p-hydroxybenzoic acid Protocatechuic acid Epi-catechin Syringic acid Catechin	Antidiabetic	In vitro and in vivo Wistar albino rats	[30]
Hydroalcoholic	Catechins Gallic acid Ferulic acid Epicatechin Chlorogenic acid	Antiproliferative Anti-inflammatory Antioxidant	In vitro HepG2 cell line	[35]
Ethanolic acetone chloroformic ethyl acetate	Phlobatannins Tannins Sterols	Antibacterial	In vitro	[29]
Methanolic	Salicylic acid p-Hydroxybenzoic acid Cathechin Vanilic acid	Antiviral	In vitro	[36]
Acetic acid	Peptides	Antimicrobial	In vitro	[37]
Water	Carbohydrates (uronic acid)	Anti-inflammatory Antinociceptive	In vivo	[39]
Toluene/ethanol	Carbohydrates (lignans)	Antioxidants	In vitro	[40]

**Table 2:** Main compounds and bioactive properties in *Z. marina*.

Extract Type	Main Compounds	Activity	Experimental Model	Reference
Methanol/water	Mixture of unsaturated fatty acids	Antioxidant Metal chelating Cytotoxic	In vitro HepG2 and S17 cel lines	[45]
Ethanolic	Pectin Zosterin	Antioxidant	In vitro	[49]
Ethanol/ Acetonitrile	Apigenin Chrysoeriol Luteolin	Antioxidant MMP-1 inhibition	In vitro Hs68 and HaCaT Cell line	[50]
Methanolic	Not reported	Antioxidant Antimicrobial	In vitro	[51]
Methanolic	Luteolin	Antiphototoxicity Antimelanogenesis	In vitro HaCaT cell line	[52]
Hrydrofilic	Zosteric acid	Antiadhesive Antiviral	In vitro	[53]
Not reported	Not reported	Antiviral	In vitro	[54]
Not reported	Rosmarinic acid Luteolin 7,3′-disulfate luteolin	Antidiabetic Antihyperlipidemic	In vitro	[55]
Ethanolic	Rosmarinic acid Zosteric acid Coumaric acid α-Eleostearic acid	Lipase inhibitory Antioxidant	In vitro	[56]
Not reported	Luteolin	Antidiabetic Antilipidemic Anti-inflammatory	In vivo	[57]
Ethanolic	Chicoric acid p-Coumaric acid Rosmarinic acid Benzoic acid Ferulic acid Caffeic acid	Anticancer	In vitro	[58]
Acetone	Diarylheptanoids Zosterabisphenone B	Anticancer	In vitro	[59]
Methanol/Toluene	Not reported	Antifungal	In vitro	[60]

**Table 3:** Main compounds and bioactive properties in *C. nodosa*.

Extract Type	Main Compounds	Activity	Experimental Model	Reference
Water	Sulphated polysaccharides (uronic acid)	Anti-obesity Lipid lowering	In vitro and In vivo Male Wistar rats	[64]
Ethanolic	Sulphated polysaccharides	Antioxidant Reducing power Antihipertensive	In vitro	[65]
Water	Sulphated polysaccharides	Antioxidant Antimicrobial Antiproliferative	In vitro	[66]
Water	Sulphated polysaccharides	Antioxidant Improve sperm quality	In vitro In vivo	[67]
Hydroalcoholic	Sulphated polysaccharides Flavonoids	Antioxidant	In vitro	[68]
Hydroalcoholic	Sinapic acid Myricetin Quercetin-3-O-rutinoside	Antioxidant	In vitro	[69]
Hydroalcoholic	Flavonoids Catechin Quercetin-3-O-rutinoside Quercetin-3-O-glucoside)	Antioxidant Protective effects on pancreatic β-cells	In vitro In vivo	[70]
Dichloromethane/ methanol	Meroterpenoid Briarane diterpene	Antibacterial	In vitro	[71]

**Table 4:** Main compounds and bioactive properties of *H. uninervis*.

Extract Type	Main Compounds	Activity	Experimental Model	Reference
Methanolic and ethanolic	Caffeic acid Chlorogenic acid Gallic acid p-Hydroxybenzoic acid	Cytotoxic Antioxidant	In vitro MCF-7 and SKOV-3 cell lines	[74]
Ethanolic	Apigenin Acacetin Coumaric acid Kaempferol Vanillic acid	Antioxidant	In vitro	[75]
Ethanolic	Tanins Flavonoids Terpenoids Saponins	Anticancer	In vitro (MDA-MB 231) cell line	[75]
Ethanolic	Unsaturated fatty acids Saturated fatty acids Flavonoids Alkanoids	Antimicrobial	In vitro	[77]
Methanolic	Not determined	Antimicrobial	In vitro	[76]
Methanolic	Not defined	Antilarvicidal Antimicrobial	In vitro	[79]
Methanolic Dichloromethane Hexane	Alkanoids Saponins Tannis Diterpenes Flavonoids Phenolic and cardiac glycosides	Antimicrobial	In vitro	[78]
Hydrophilic	Not determined	Antilarvicidal	In vitro	[79]
Ethyl acetate	Not determined	Antilarvicidal	In vitro	[80]
Ethyl acetate	Alkaloid Flavonoids Terpenoids Phenols Quinones Steroids	Anticancer	In vitro HT29, HeLa, A549, A375 cell line	[83]
Ethanolic Ethyl acetate	Flavonoids Saponins Esteroids Triterpenoids	Antidiabetic	In vitro	[81]

**Table 5:** Main compounds and bioactive properties in *T. hemprichii*.

Extract Type	Main Compounds	Activity	Experimental Model	Reference
Endophytic fungi extracts	Not determined	Antimicrobial	In vitro	[86]
Acetone Ethanolic	Not determined	Antimicrobial	In vitro	[88]
Methanolic	Isoscutellarein 7-O-β-xylopyranoside-2′′-O-sulfate Isoscutellarein 7-O-β-xylopyranoside Isoscutellarein	Antimicrobial	In vitro	[90]
Methanolic	Thalassiolins A–C	Antiviral	In vitro	[91]
Methanolic	Luteolin 7-*O*-β-d-glucopyranosyl-2”-sulfate	Antibiotic	In vitro	[92]
Methanolic and ethanolic	Tannins Flavonoids Saponins Esteroids	Antimicrobial	In vitro	[94]
Ethanolic	Thalassiolins A, B, C, D Phenolic metabolites	Antioxidant	In vitro	[95]
Ethanolic	Phenolic metabolites	Antidiabetic	In vitro and in vivo Wistar albino rats	[96]
Ethanolic Methanolic	Non determined	Larvicidal	In vitro	[97,98]

**Table 6:** Main compounds and bioactive properties of *H. stipulacea*.

Extract Type	Main Compounds	Activity	Experimental Model	Reference
Ethanolic	Phenolics Flavonoids Diterpenes	Antidiabetic	In vitro and in vivo Wistar albino rats	[96]
Ethanol/Water	Carotenoids Carbohydrates	Antioxidant	In vitro WI-38 cell line	[105]
Hexane Ethyl acetate	Luteolin Apigenin Matairesinol	Anticancer	In vitro SH-SY5Y, HCT-116, MG-63, HepG2, hCMEC/D3 cell lines	[106]
Ethyl acetate Methanolic	Cirsimarin Spiraeoside Amino acids (N-acetyl-L-tyrosine)	Lipid-reducing	In vivo Zebrafish larvae	[106]
Hexane Methanolic	Fatty acids (13-decosenamide) Cinnamic acids	Antifouling	In vitro and in vivo Mussel larvae	[106]
Ethanolic	Carbohydrates Phenolics Flavonoids Terpenoids	Antibacterial	In vitro	[108]
Ethanolic	Carbohydrates Phenolics Flavonoids Terpenoids	Anti-inflammatory	In vivo Wistar albino rats	[108]
Aqueous Hydroalcoholic Chloroformic Ethyl acetate	Non determined	Antimicrobial	In vitro	[109]
Methanolic Chloroform/methanol	P-hydroxybenzoic acid Bis (2-ethyl hexyl) phthalate Benzoic acid P-Hydroxybenzaldehyde Thymidine Stigmasterol Oleic acid Linoleic acid Linoleic methyl ester Apigenin	Antiosteoclastogenic AntioxidantCytotoxic	In vitro	[110]
